# Succinylation Inhibits the Enzymatic Hydrolysis of the Extracellular Matrix Protein Fibrillin 1 and Promotes Gastric Cancer Progression

**DOI:** 10.1002/advs.202200546

**Published:** 2022-07-28

**Authors:** Xingyun Wang, Xiao Shi, Hongcheng Lu, Chen Zhang, Xiang Li, Tiancheng Zhang, Jiajia Shen, Jianfei Wen

**Affiliations:** ^1^ Department of General Surgery First Affiliated Hospital of Nanjing Medical University Nanjing 210029 China; ^2^ Hongqiao International Institute of Medicine Tongren Hospital Shanghai Jiao Tong University School of Medicine No. 1111, XianXia Road Shanghai 200336 China; ^3^ Department of Gastroenterology Zhongda Hospital School of Medicine Southeast University Nanjing 210009 China; ^4^ Department of Urology Zhongda Hospital Affiliated to Southeastern China University Nanjing 210029 China; ^5^ Department of Biotherapy Medical Center for Digestive Diseases Second Affiliated Hospital of Nanjing Medical University Nanjing 210011 China; ^6^ Department of Surgical Oncology Jiangsu Province Hospital of Chinese Medicine Affiliated Hospital of Nanjing University of Chinese Medicine Nanjing 210029 China

**Keywords:** fibrillin 1, gastric cancer, MMP2, succinylation, TGF‐*β*1

## Abstract

Extracellular matrix (ECM) remodeling is crucial in the regulation of gastric cancer (GC) progression. This work aims to reveal novel posttranslational modifications and their relevant mechanisms in GC. In 3D matrix culture and animal models, it is found that fibrillin 1 (FBN1) expression is increased in advanced GC and has succinylation modification. The succinylation modification of FBN1 blocks its degradation by matrix metalloproteinases (MMPs). The long‐term accumulation and deposition of FBN1 enhance tumor progression by activating TGF‐*β*1 and intracellular PI3K/Akt pathway. The FBN1 succinylation site monoclonal antibody can effectively intervene the effect of succinylation modification and inhibit GC progression. FBN1 is specifically upregulated in the progression of GC compared with other tumors. In conclusion, FBN1 is widely present in the form of K672‐succinylated modifications in GC. Besides, the succinyl group of FBN1 blocks its binding to MMP2, inhibits its degradation by MMP2, and leads to the accumulation of FBN1, which poses a long‐term risk to the poor prognosis of GC.

## Graphical abstract

Gastric cancer (GC) has severely high morbidity and mortality rates worldwide, and abnormal expression and degradation of the extracellular matrix (ECM) affect the occurrence and outcome of GC. This work is focused on how fibrillin 1 (FBN1), a primary component of the ECM, and its modified substructure affect GC development.

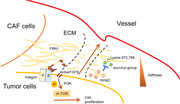

## 1 Introduction

The extracellular matrix (ECM) is critical for tumor initiation and development.^[^ ^1^, ^2^ ^]^ This has been confirmed in mechanistic studies on tumorigenesis and development related to ECM remodeling.^[^ ^3^, ^4^ ^]^ For example, dynamic ECM changes in the microenvironment affect the occurrence and progression of breast cancer. Furthermore, the ultrasonic density of stromal tissue can serve an index for tumor prognoses.^[^ ^5^ ^]^ Previous studies confirmed that cancer‐associated fibroblasts (CAFs) and tumor cells support tumor cell growth and metastasis through an amino acid exchange in the ECM.^[^ ^1^ ^]^ Thus, tumorigenesis leads to drastic changes in the physicochemical properties of the ECM.^[^ ^6^ ^]^ Furthermore, structural alterations to ECM proteins may influence their role in gastric cancer (GC).^[^ ^7^ ^]^ Therefore, understanding the relationships between ECM proteins and tumor progression may improve clinical tumor diagnoses and interventions.

GC has severely high morbidity and mortality rates worldwide,^[^ ^8^, ^9^ ^]^ and abnormal protein expression and degradation of the ECM affect the occurrence and outcome of GC.^[^ ^10^, ^11^ ^]^ ECM proteins produce adverse biological effects by combining with various active proteins, cytokines, and extracellular receptors in the tumor. Importantly, abnormal matricellular protein expression and modifications are specific and sensitive enough for disease diagnosis,^[^ ^12^, ^13^ ^]^ and targeting matrix metalloproteinases (MMPs) may interfere with tumor progression.^[^ ^14^ ^]^ However, several ECM proteins are found around normal cells, not only tumor cells, complicating the clinical diagnosis.^[^ ^15^ ^]^ Furthermore, preclinical therapy studies that targeted ECM proteins and assessed the effects on tumor progression concluded that such therapies were not feasible; the risk of tumor proliferation and metastasis was too high.^[^ ^16^ ^]^ Nonetheless, phosphorylation sites, possibly even a protein substructure, could be a biomarker for tumor diagnosis and target for intervention.^[^ ^17^ ^]^

Adequate diagnostic biomarkers for GC are lacking, but protein substructure studies may provide a solution to this problem. Previously, our laboratory focused on howLDHA and its modified substructure affected GC development.^[^ ^18^ ^]^ FBN1 is the primary component of microfibrils, the ECM skeleton, and the ECM of elastic and inelastic tissues. It also has essential roles in tissue development and organization, including blood vessels, bones, limbs, and eyes.^[^ ^19^ ^]^ The Cancer Genome Atlas (TCGA) data illustrated that high FBN1 expression negatively correlated with the survival of patients with GC. Similar to other ECM‐related proteins, FBN1 is highly expressed in tumors. The succinylation modification is a specific protein modification type mediated by succinyl‐CoA, whereby a succinyl group binds to the lysine site of a protein.^[^ ^20^ ^]^ Interestingly, tumor tissues widely express succinylated FBN1 compared with normal tissues. Because many of these modified structures are concentrated in tumor tissue, they might be critical for GC.^[^ ^21^ ^]^ Therefore, this study explored the function of FBN1 and its substructure in GC development and progression.

## 2 Results

### 2.1 FBN1 is Highly Expressed in GC and Closely Associated with Patient Survival

**Figure 1:**
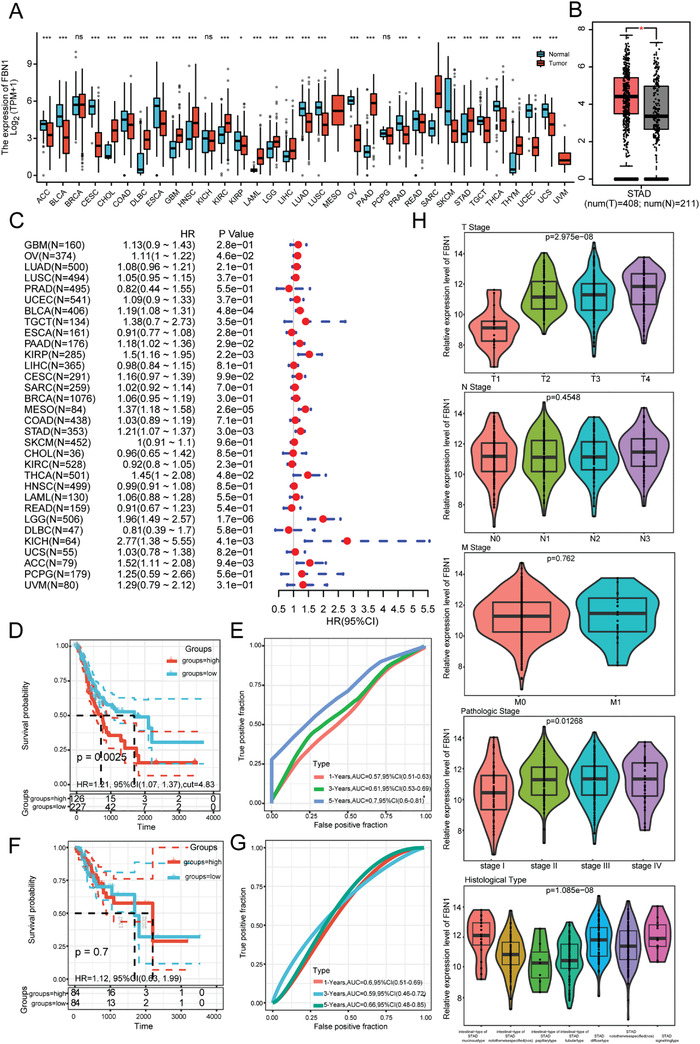
FBN1 is highly expressed in gastric cancer (GC) but not in other cancers. A) FBN1 mRNA expression in GC and adjacent normal tissues from The Cancer Genome Atlas (TCGA) datasets. Mann–Whitney U Test was used for statistical analysis. B) Analysis of unpaired samples from the GEPIA database shows the FBN1 expression in GC (*n* = 408) and normal tissues (*n* = 211). C) Bioinformatics analysis of the correlation between FBN1 and prognosis of various tumors. D,F) Kaplan–Meier analysis of the overall survival (OS) of patients with GC based on FBN1 expression in all patients (D) and in stages T1–T2 (F). E,G) ROC curves of the accuracy and reliability of FBN1 in predicting the 1‐, 3‐, and 5‐year outcomes of GC in all patients (E) and in stages T1–T2 (G). AUC greater than 0.7 has certain accuracy. H) Plural online databases were used in the analysis of the correlation of the relative expression level of FBN1 with TNM stage, pathological stage, and histological type. Data are shown as mean ± SD, *p < 0.05, **p < 0.001, ***p < 0.0001.

Our bioinformatic analysis demonstrated a remarkable difference in FBN1 expression among multiple tumor types (**Figure** **1**A). Specifically, FBN1 expression was significantly higher in GC compared to normal tissue (Figure 1B). Furthermore, we analyzed 32 tumor types prognostically, finding that the hazard ratio (HR) of patients with GC expressing FBN1 was as high as 1.21, which ranked higher on a scale (Figure 1C). High FBN1 expression was significantly related to poor outcomes in advanced GC (*p* < 0.05, Figure 1D,E). However, the survival analysis of all GC patients did not identify differences in FBN1 expression and the prognosis (Figure 1F,G). FBN1 expression was upregulated in tumor (T) stages T1–T4, especially stages T2–T4 (Figure 1H), but did not differ among the node and metastasis stages. FBN1 was highly expressed in all pathological and histological stages. Together, these results confirm a relationship between high FBN1 expression and survival for patients with GC.

### 2.2 FBN1 is Highly Succinylated at Sites K672 and K799 in GC

**Figure 2:**
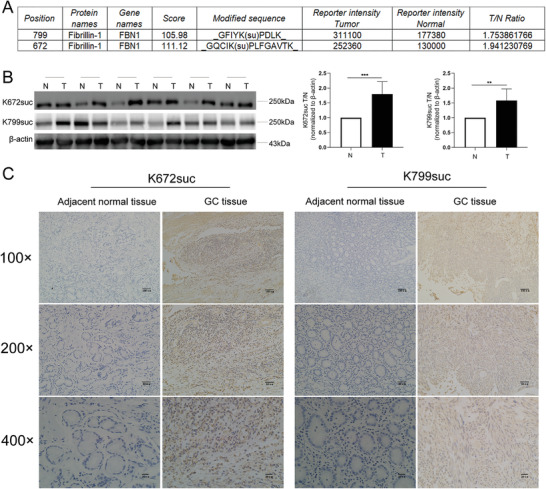
FBN1 is succinylated at K672 and K799 in gastric cancer (GC). A) Mass spectrometry analysis of the succinylated FBN1 peptides of GC tissues. B) Western blot analysis of the protein levels of GC (*n* = 6) and adjacent normal tissues (*n* = 6) by two kinds of succinylation‐specific antibodies. C) Immunohistochemical analysis of K672‐ and K799‐succinylated FBN1 in GC and adjacent normal tissues. Data are shown as mean ± SEM, *p < 0.05.

First, we extracted total protein from tissues of seven patients with GC. Then, we assayed for enriched succinylation modification‐related proteins using antibodies against the succinylated forms of the sites K672 and K799. In addition, high‐throughput mass spectrometry was used to analyze succinylation modification‐related protein expression in GC and normal tissues. We found that two lysine‐succinylated modification sites in FBN1, namely K672 and K799, were upregulated by 1.94 and 1.75 times, respectively (**Figure** **2**A). Next, we detected the FBN1‐K672suc and FBN1‐K799suc protein levels by Western blotting using antibodies against the succinylated forms of K672 and K799 (Figure 2B). The results showed that these levels were markedly higher in GC than in normal tissues, consistent with the mass spectrometry results. Finally, we performed immunohistochemical analyses using tissue microarrays, two customized antibodies, and the tissues of patients with GC to investigate the role of succinylated FBN1 in GC. The succinylated FBN1 level was higher in GC than in normal tissues (Figure 2C).

### 2.3 FBN1 Succinylation at K672 Affects its Binding to MMP2 in the Tumor Microenvironment

**Figure 3:**
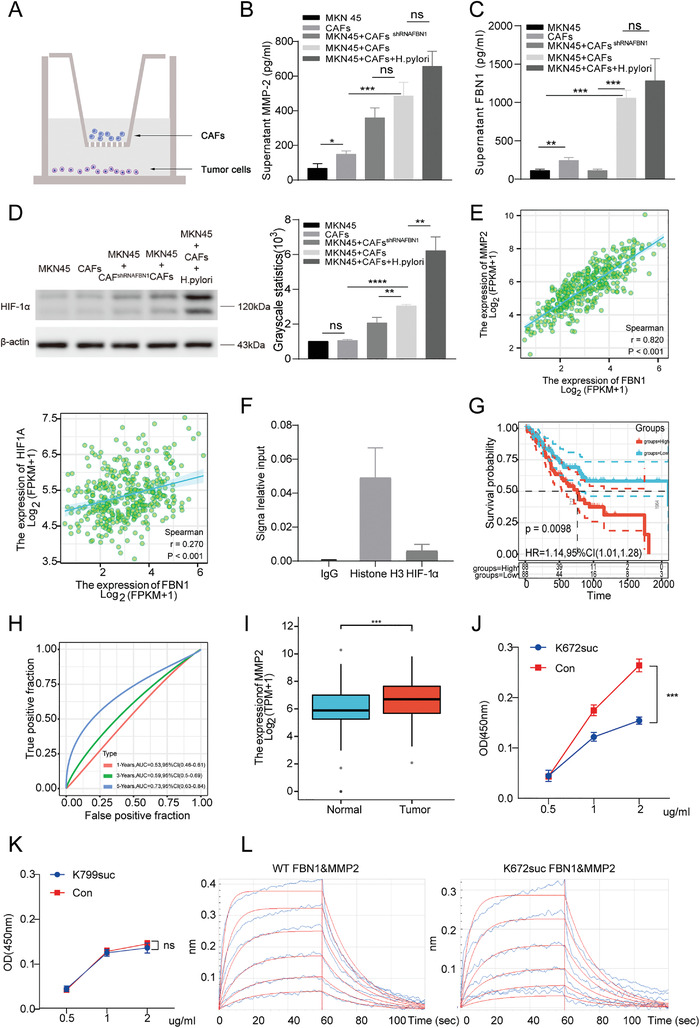
Succinylation inhibits the binding of MMP2 to FBN1. A) Schematic diagram of the co‐culture system. B,C) Enzyme‐linked immunosorbent assay (ELISA) of the levels of MMP2 (B) and FBN1 (C) in the supernatant of each group (*n* = 3). D) Western blot analysis of HIF‐1*α* protein levels in each group. E) Correlation analysis between FBN1 and MMP2 or HIF‐1*α* in gastric cancer (GC). Data were from The Cancer Genome Atlas (TCGA) datasets. F) chromatin immunoprecipitation (ChIP) assay of MKN45 cells cultured with cancer‐associated fibroblasts (CAFs). Anti‐HIF‐1*α* immunoprecipitated the DNA fragments of MMP2. G) Kaplan–Meier analyses of the overall survival (OS) of patients with GC according to MMP2 levels. Data were from TCGA datasets. H) ROC curve analysis of the accuracy and reliability of MMP2 in predicting the 1‐, 3‐, and 5‐year prognoses of patients with GC by ROC curve. AUC > 0.7 has certain accuracy. I) MMP2 levels in GC and normal tissues determined by TCGA datasets. J,K) ELISA of the binding of K672‐succinylated FBN1 (J) and K799‐succinylated FBN1 (K) to MMP2. L) Biolayer interferometry (BLI) analysis of the interaction of wild‐type (WT) FBN1 polypeptide and succinylated K672 polypeptide with MMP2. Data are shown as mean ± SEM, *p < 0.05, **p < 0.01, and ***p < 0.001. Experiments were repeated three times.

We established a method of co‐culture between CAFs and GC tumor cells (MNK45); the tumor cells were plated at the bottom of the culture plate, while CAFs were plated in a small polyvinylidene difluoride membrane chamber (pore size, 0.4 µm) and hung above the tumor cells (**Figure** **3**A). Next, we collected the co‐culture supernatant and tumor cell lysate, and detected MMP2 in the culture medium using an enzyme‐linked immunosorbent assay (ELISA). The free MMP2 level in the MKN45–CAF co‐culture group was considerably higher than that in tumor cells or CAFs alone. In contrast, the free MMP2 expression level in the CAF^ShRNAFBN1^ treatment group decreased minimally.

We also created a *Helicobacter pylori*‐positive co‐culture group where the MMP2 expression level was consistent with the MKN45–CAF co‐culture group (Figure 3B). FBN1 expression was also detected and found to be substantially upregulated in the co‐culture group. However, it decreased after short hairpin (sh)RNA interference (Figure 3C).

Western blotting of tumor cell lysates indicated that hypoxia‐inducible factor 1*α* (HIF‐1*α*) protein expression was considerably upregulated in the MKN45–CAF co‐culture compared with MKN45 cells alone. A decreased FBN1 level reduced the HIF‐1*α* level, which was highly expressed in the hypoxic *H. pylori*‐positive group (Figure 3D).

Protein succinylation is closely related to protein degradation, and FBN1 degradation is attributed to the MMP family. Therefore, MMP2 is important in GC progression. We found a strong correlation between FBN1 and MMP2 expression through a bioinformatic analysis (Figure 3E). Moreover, we identified a positive correlation between HIF‐1*α* and FBN1 expression. To further investigate the relationship between HIF‐1*α* and MMP2, we used chromatin immunoprecipitation quantitative polymerase chain reaction (qPCR), which verified the transcriptional regulation of MMP2 by the transcription factor HIF‐1*α* (Figure 3F). A luciferase reporter assay obtained the same results (Extended Figure 1). We found that HIF‐1*α* promoted MMP2 transcription after it was downregulated; the qPCR analysis for MMP2 gene expression showed that HIF‐1*α* promoted MMP2 transcription.

We found that increased MMP2 expression was linked to poor outcomes in patients with GC (Figure 3G,H). Further, analysis of TCGA database identified high MMP2 expression in tumor tissues (Figure 3I). Therefore, we used wild‐type (WT) and mutated K672 and K799 and assessed, using an ELISA, their abilities to bind MMP2 (Figure 3J,K). We found that the binding of FBN1 to MMP2 protein decreased considerably after succinylation, suggesting that this modification inhibited their binding. The co‐immunoprecipitation assay results showed that FBN1 interacted with MMP2 (Extended Figure 2). Subsequently, biolayer interferometry was performed to determine the binding strengths of FBN1 and succinylated FBN1 to MMP2. The results confirmed that the unmodified polypeptides bound MMP2 more strongly than the modified polypeptides, suggesting that succinylation could block the binding of MMP2 to FBN1 (Figure 3L).

### 2.4 Succinylated K672 FBN1 Predicts Survival and Outcomes of Patients with GC, Causes MMP2 Accumulation, and Promotes Stromal Degradation and Tumor Migration

**Figure 4:**
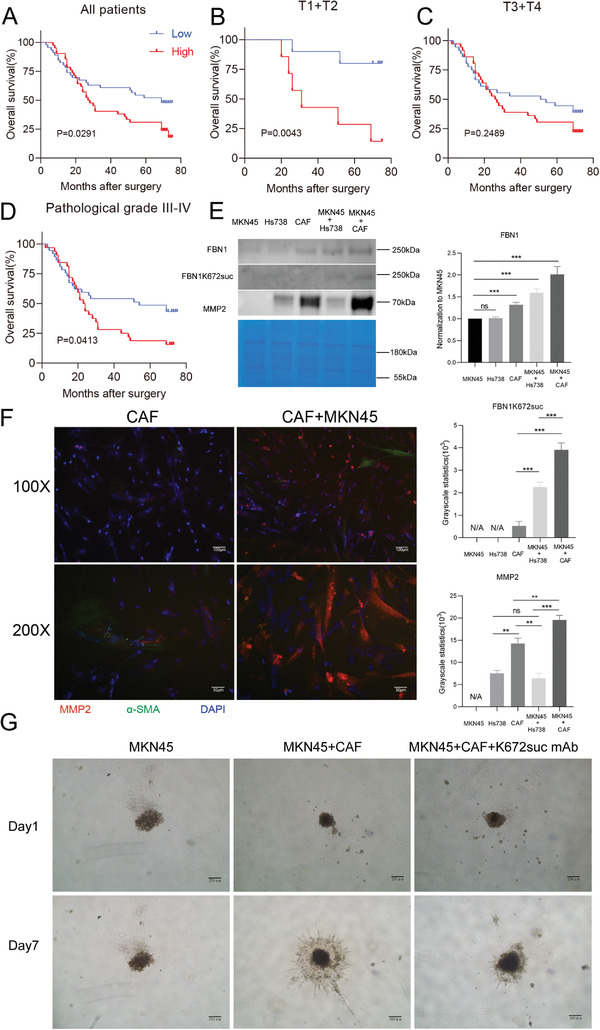
Excessive MMP2 causes stromal degradation and promotes tumor metastasis. A–D) Kaplan–Meier analysis of the overall survival (OS) of patients with gastric cancer (GC) according to succinylated K672 FBN1 expression in all patients (A), patients in stages T1–T2 (B), patients in stages T3–T4 (C), and patients with pathological grades III–IV (D). E) The expression levels of FBN1, succinylated K672 FBN1, and MMP2 in the indicated groups were determined by Western blot. Coomassie brilliant blue staining results were used as the control. F) Immunofluorescence assay for the detection of *α*‐SMA (green) and MMP2 (red) in cancer‐associated fibroblasts (CAFs) treated with or without MKN45 cells. G) 3D spheroid invasion assays of the invasive ability of MKN45 in different indicated treatments.

To verify whether succinylated FBN1 could predict tumor prognosis, we performed a tissue microarray analysis that included approximately 150 patients using an FBN1‐K672suc monoclonal antibody. We scored and compared normal and cancer tissues, and found that the overall survival (OS) was shorter for patients with a high level of succinylated FBN1 than those with a low level (**Figure** **4**A). Furthermore, OS differed between the high and low succinylation groups at stages T1 and T2 (Figure 4B) as well as between pathological staging grades III and IV (Figure 4D). On the other hand, patients in stages T3 and T4 did not show divergent results (Figure 4C).

We next constructed co‐culture systems of MKN45 with Hs738 cells (normal gastric cells) or CAFs and collected the culture supernatants to explore succinylated FBN1 production in the GC microenvironment. We used a 100‐kDa centrifugal filter unit to concentrate the proteins and then analyzed the FBN1, FBN1‐K672suc, and MMP2 protein levels using Western blotting (Figure 4E). FBN1, FBN1‐K672suc, and MMP2 levels were higher in the MKN45–CAF co‐culture group supernatant than in the supernatants of other groups. Furthermore, the immunofluorescence assay demonstrated that MMP2 expression in the CAF–MKN45 co‐culture group increased after 72 h of co‐culture compared to the CAF alone group (Figure 4F). Finally, we performed a 3D spheroid invasion assay (Figure 4G). The sphere was cultured for 7 days, after which the invasion channel of tumor cells was apparent after adding CAFs compared with MKN45 cells alone. We also used the FBN1‐K672suc monoclonal antibody to interfere with the 3D spheroid, finding that invasion of the sphere into the surrounding matrix was remarkably weakened.

In summary, cross‐talk between tumor cells and CAFs increased succinylated FBN1 and MMP2 expression and promoted tumor cell invasion into the stroma.

### 2.5 Abundant FBN1 in the Intercellular Stroma Promotes Tumor Proliferation Through the Phosphoinositide 3‐Kinase (PI3K)/Protein Kinase B (Akt) Signaling Pathway

**Figure 5:**
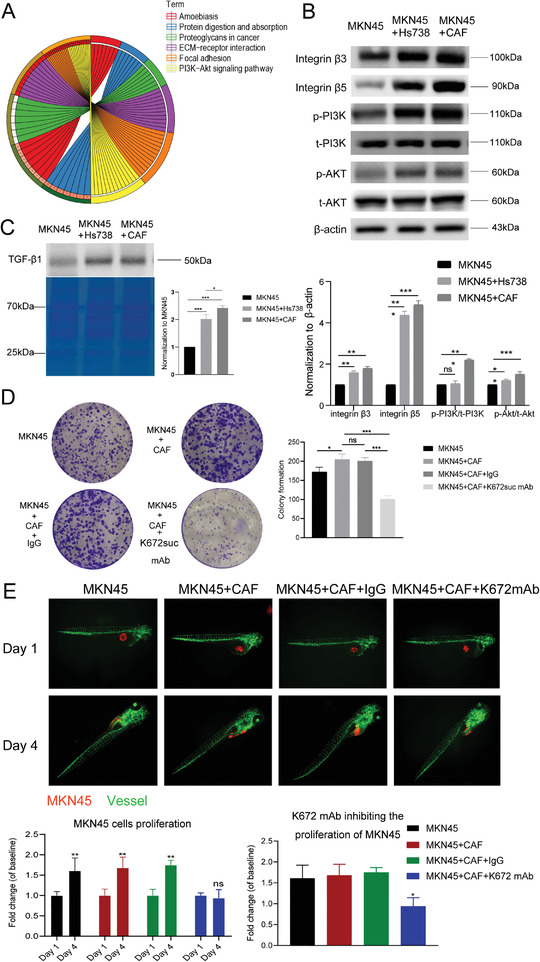
Abundant FBN1 in the stroma promotes tumor proliferation through the LTBP1–integrin–TGF–*β*1 pathway. A) KEGG enrichment analysis of FBN1‐related signaling pathways. B) Integrin *β*3, integrin *β*5, and PI3K/Akt signal pathway protein levels in MKN45, MKN45 with Hs738, and MKN45 with cancer‐associated fibroblast (CAF) groups. C) Detection of TGF‐*β*1 protein level in indicated groups. Coomassie brilliant blue staining results were used as the control. D) Proliferation of MKN45 cells analyzed by plate colony formation assays. E) Zebrafish cell line‐derived xenograft (zCDX) assays of the proliferation of MKN45 cells in the indicated four groups. The cells were separately treated with IgG and K672suc monoantibody (*n* = 300).

Our bioinformatic analysis identified a relationship between FBN1 expression and tumor proliferation, and the PI3K/Akt signaling pathway (**Figure** **5**A). We used cell lysates from the MKN45–CAF system co‐cultured for 72 h to assess the PI3K/Akt signaling pathway. We found that the supernatant was rich in FBN1, and the intracellular PI3K/Akt signaling pathway was continuously activated in tumor cells (Figure 5B). MKN45 cells with or without CAFs were interfered by MMP2 and PI3K was inhibited. These results showed that tumor growth was inhibited by suppressing PI3K and MMP2 (Extended Figure 3). Furthermore, these samples were used to detect integrin *β*3 and *β*5 expression. The results showed that excitation of the intracellular activation signal was related to these integrins. Also, the expression of activated transforming growth factor *β*1 (TGF‐*β*1) was increased in the culture supernatant (Figure 5C).

We also performed a plate cloning assay in the same co‐culture system to directly confirm that FBN1 promotes tumor cell proliferation. The proliferation of cells in the MKN45–CAF co‐culture was remarkably greater than that of the cells in the other cultures. Interestingly, tumor cell proliferation decreased considerably in the group treated with the anti‐FBN1‐K672suc monoclonal antibody (Figure 5D). The cell invasion assay showed that this antibody reduced the infiltration of MKN45 cells into the matrix gel (Extended Figure 4). Subsequently, the MKN45–CAF co‐culture system was applied to the zebrafish cell line‐derived xenograft model to better illustrate the relationship between FBN1 and tumor proliferation(Figure 5E). The results were consistent with the plate cloning experiment. Together, these results suggest that FBN1 promotes GC cell proliferation, and our FBN1‐K672suc monoclonal antibody is a promising tumor proliferation intervention tool.

## 3 Discussion

ECM‐related proteins are important in tumorigenesis and tumor development.^[^ ^22^ ^]^ Thus, current research is focused on anti‐tumor therapies involving the ECM.^[^ ^16^ ^]^ The current study found that FBN1 expression in various tumor tissues differed from that in normal tissues, and it was notably higher in GC tissues. Furthermore, mass spectrometry confirmed a high FBN1 succinylation level, and the OS analysis of patients with GC determined that succinylated FBN1 was a sensitive predictor of poor prognosis. MMP2 is an effector molecule that also leads to a poor prognosis in GC, and we confirmed that the MMP2 level positively correlated with FBN1 upregulation. Moreover, FBN1 accumulation led to local tissue stiffness, increasing the HIF‐1*α* level. Thus, we evaluated the influence of HIF‐1*α* on the DNA binding of the MMP2 promoter using a ChIP‐qPCR assay. The result suggested that MMP2 overexpression was derived from HIF‐1*α* transcription. The mechanical interaction between the accumulated FBN1 and integrins *β*3 and *β*5 on the tumor cell surface resulted in the separation of TGF‐*β*1 from the latency‐associated peptide complex, activating TGF‐*β*1 and triggering the PI3K/Akt signaling pathway, thereby promoting tumor proliferation.

The functional mechanism of FBN1 in tumors has rarely been studied, but some researchers hypothesize that FBN1 participates in tumorigenesis and carcinoma development.^[^ ^23^ ^]^ The present study found that FBN1 differs from many tumor‐related proteins. In most tumors, we found no remarkable differences in FBN1 expression. However, in GC, we observed remarkably higher FBN1 expression, indicating that it is tissue‐specific.^[^ ^24^ ^]^ Usually, FBN1 maintains the normal physiology of the organism.^[^ ^25^ ^]^ However, the mechanism of action of FBN1 in GC is unknown. Therefore, future studies will need to explore new substructures and functions resulting from the modification of these proteins to explain their roles in a healthy body and pathological processes.^[^ ^26^, ^27^ ^]^

In our specific modification proteomics study, we identified two succinylation modification sites in FBN1. Consequently, we explored the roles of succinylated FBN1 substructures in GC occurrence and development. Succinylation modification means that a succinyl group is bound to a lysine group by succinyl‐CoA under certain physical and chemical conditions. The lysine site, which has a positive charge, changes to a negative charge.^[^ ^23^ ^]^ Our high‐throughput mass spectrometry data indicated that FBN1 has two succinylated substructures. High FBN1 expression in GC also provides evidence that the succinylated FBN1 structure strongly regulates the pathological process of GC. Interestingly, FBN1 expression is abundant in normal tissues, whereas succinylated FBN1 is more common in GC tissues, suggesting that the function of FBN1 may be relatively independent of succinylated FBN1 in GC. The substructure produced by the succinylation modification is likely to give new and distinct functions to WT FBN1. Therefore, we suggest that a clear description and understanding of the function of this substructure may be an important means to combat the carcinogenic mechanisms and, thus, the development of GC.

We also found a relationship between FBN1 expression and the survival time of patients with GC, though this result only applied to patients in the primary stage. While higher FBN1 expression corresponded to a shorter survival time in primary stage GC patients, no differences were identified between the FBN1 levels and outcomes in patients with advanced‐stage GC. Consequently, we designed a succinylated FBN1 antibody and applied it to the survival and prognosis analysis of patients with GC. A high level of succinylated FBN1 expression in patients with primary stage disease can reliably indicate that these patients may face a very poor prognosis. However, we could not evaluate survival predictions for patients with advanced‐stage disease because of the reduced chance of surgery and the rapidly developing tumor.

In summary, the expression level of the FBN1 substructure is more sensitive for judging tumor prognosis than the pan‐FBN1 expression level. Identifying the modification site and using it as a therapeutic target allows us to interfere with tumor rather than normal tissues as specifically as possible by intervening with the succinylation‐specific modification of FBN1. Doing so can reverse a series of complex challenges after FBN1 succinylation modification. Therefore, targeted inhibition of the FBN1‐specific succinylation modification may become a new anti‐GC clinical strategy.

In the 3D matrix invasion and zebrafish experiments we found that antibodies against the K672 site of FBN1 effectively inhibited tumor invasion and proliferation. During succinylation modification, lysine binds to the succinyl group, which changes the local potential. The reason for selecting a monoclonal antibody is that its binding does not cause a charge change but prevents the binding of the K672 site of FBN1 to a succinyl group.^[^ ^28^ ^]^

MMP2 is a functional protein in the ECM that degrades other ECM and extracellular proteins.^[^ ^29^ ^]^ MMP2 is also an ECM‐associated protein linked to GC development.^[^ ^30^ ^]^ In GC, the expression levels of MMP2 are higher than those of other proteins from the same family.^[^ ^31^ ^]^ Our study identified a positive association between ECM protein deposition and the extensive level of endogenous free MMP2. We suspect that free MMP2 overexpression in GC is related to local tissue stiffness because MMP overexpression is usually accompanied by tissue stiffness.^[^ ^32^ ^]^ This mechanism is hypothesized to produce HIF‐1*α* in a hypoxic environment caused by local stiffness. Moreover, HIF‐1*α* is a versatile transcription factor. Our ChIP experiments confirmed that high levels of free MMP2 are derived from HIF‐1*α* transcription in GC. Notably, our previous studies showed that *H. pylori* infection increases the HIF‐1*α* levels in GC.^[^ ^33^ ^]^ In the present study, HIF‐1*α* was also increased after *H. pylori* infection and accelerated the increase of free MMP2. These findings suggest that treating *H. pylori* infections is another important link that cannot be ignored during GC interventions.

Our study demonstrated that the CAF–MKN45 co‐culture system enriched MMP2 in the cell culture supernatant. Increased levels of MMP2 proteins degrade the matrix and collagen tissues in the intercellular stroma; this opens up a channel for the infiltration and migration of tumor cells, which invade the basement membrane and enter the circulatory system.^[^ ^34^ ^]^ Our bioinformatics analysis showed a positive correlation between MMP2 expression and FBN1, which was verified using BLI experiments; these results demonstrated that FBN1 interacts with MMP2. Interestingly, the interaction strength between succinylated FBN1 and MMP2 was much lower than that between WT FBN1 and MMP2, indicating that the succinylation modification inhibited FBN1 degradation by MMP2. This factor may also explain the limited effectiveness of anti‐MMP2 therapies.

The excessive upregulation of ECM proteins, such as FBN1, leads to stiffness of the local ECM structure.^[^ ^35^, ^36^ ^]^ Integrins *β*3 and *β*5 are transcellular signal receptors and, in tumors, they bind to the latent TGF‐*β* binding protein structure of inactivated TGF‐*β*1 protein, which is related to TGF‐*β*1 activation.^[^ ^37^ ^]^ A stiff ECM coordinates with integrins *β*3 and *β*5 to produce a mechanical force that promotes dissociation of the latent TGF‐*β*1 complex, thus causing the release of a large amount of TGF‐*β*1. This massive activation of TGF‐*β*1 facilitates tumor cell proliferation and migration.^[^ ^38^ ^]^ TGF‐*β*1 has a regulatory effect on immune cells^[^ ^39^ ^]^ and can activate many tumor‐related signaling pathways, such as the mitogen‐activated protein kinase and PI3K/Akt pathways.^[^ ^40^, ^41^ ^]^ The tumor‐promoting effects of TGF‐*β*1, including effector lymphocyte regulation and angiogenesis, have brought more obstacles to tumor therapy.^[^ ^42^, ^43^ ^]^ Our bioinformatics analysis found a strong correlation between FBN1 and the PI3K/Akt signaling pathway in GC. The succinylation modification leads to FBN1 accumulation, which interacts with the latent TGF‐*β*1 complex and gives a downstream focal adhesion kinase adaptor protein signal, directly promoting activation of the PI3K/Akt signaling pathway. Therefore, in GC with highly succinylated FBN1 expression, we hypothesize that extracellular components allow tumor cells to increase the Akt activation signals, resulting in tumor proliferation.

## 4 Conclusions

The abnormal accumulation of succinylated FBN1 leads to the upregulation of extracellular factors, such as MMP2, and promotes various responses, such as the intracellular proliferation signals of tumor cells. These events increase the risk of a poor prognosis for patients with GC. Furthermore, we demonstrated that targeting the ECM and interfering with posttranslational modification processes of tumor‐related proteins may be an important direction for anti‐tumor therapy development.

## 5 Experimental Section

### Human GC Tissue Specimens

Primary gastric adenocarcinoma samples were obtained from patients hospitalized in the Second Affiliated Hospital of Nanjing Medical University who were treated by surgery only, without any other treatments before surgery, and were obtained after informed consent. This study was approved by the Ethics Committee of the Second Affiliated Hospital of Nanjing Medical University.

### Immunohistochemical Analysis

The succinylated FBN1 peptides, PKGFIYK (succ) PDLKTC‐NH2 and GQCIK (succ) PLFGAVTK‐NH2, were synthesized and used to prepare rabbit polyclonal antibodies from ChinaPeptides Co., Ltd. (Shanghai, China). Seven pairs of GC and adjacent normal tissues were treated with 4% paraformaldehyde and embedded in paraffin. Then, the slides were prepared using 2 µm‐thick continuous sections. The slides were roasted at 65 °C for 2 h and then dewaxed with xylene three times for 5 min. The slides were soaked in 95%, 90%, 85%, 75%, 60%, and 50% anhydrous ethanol for 5 min successively and then washed with PBS several times. Antigen repair was performed with 1 × 10^−3^ m EDTA solution (pH 8.0) for 90 s at 110 kPa. FBN1 antibody (Thermo Fisher Scientific, Cat# MA5‐12770, RRID: AB_10988544) was diluted to 1:200, and K672‐succinylated FBN1 (FBN1‐K672suc) and K799‐succinylated FBN1 (FBN1‐K799suc) antibodies were diluted to 1:200.

Multiplication of staining intensity and the percentage of tumor‐positive cells were calculated as immunochemical staining indexes. According to the staining intensity, the scores of 0, 1, 2, and 3 indicate no staining, weak staining, moderate staining, and strong staining, respectively. The percentages of positive cells of <10%, 10%–30%, 31%–50%, and >50% were recorded as 0, 1, 2, and 3, respectively. The seventh edition of the Union for International Cancer Control and the American Joint Committee on Cancer TMN staging system was used to classify the samples. Each experiment was replicated three times.

### Cell Culture and Treatment

MKN45 (RRID: CVCL_0434) and Hs 738.St/Int (RRID: CVCL_0880) cells were purchased from American Type Culture Collection (Manassas, VA, USA), cultured with Dulbecco's modified Eagle's medium (DMEM; Invitrogen, Carlsbad, CA, USA) containing 10% FBS (Gibco, Grand Island, NY, USA) and 1% penicillin/streptomycin (Thermo Scientific, Waltham, MA, USA), and maintained in a 5% CO2 incubator at 37 °C. Human gastric noncancerous mucosal tissues were collected from the same patients at least 5 cm from the outer edge of the tumor according to the reported protocol.^[^ ^44^ ^]^In brief, the samples were washed with serum‐free DMEM, cut into small pieces, and incubated with 0.15% collagenase IV solution at 37 °C for 40 min. The digested cells were filtered through a 40 µm cell strainer (MILEX‐GP) and centrifuged at 1500 rpm for 10 min. Single cell suspension was cultured in a fibroblast culture medium kit (Cat. No. P60108. Innoprot) for 24 h, and the fibroblasts were attached to the culture plate. After 24 h of culture, the unattached cells were removed, and the adherent cells were further cultured for the experiment. In the experiment, CAFs with less than five generations were used. The experiments were replicated three times.

### Western Blot

Total protein from GC and normal tissues or cultured cell samples were lysed with a radioimmunoprecipitation assay lysis buffer with protease inhibitor cocktails (Roche Applied Science, Penzberg, Germany) and then centrifuged. The supernatant was collected and boiled for 8 min with sodium dodecyl sulfate–polyacrylamide gel electrophoresis sample‐loading buffer and then, fractionated and transferred to polyvinylidene difluoride membrane (Millipore, Bedford, MA, USA). Subsequently, the membrane was blocked with 5% BSA and incubated with the following primary antibodies: FBN1 antibody (Thermo Fisher Scientific, #MA5‐12770, RRID: AB_10988544), anti‐integrin beta 3 antibody (Abcam, ab179473), anti‐integrin beta 5 antibody (Abcam, ab184312), anti‐PI3 kinase catalytic subunit gamma/PI3K‐gamma antibody (Abcam, ab139307), anti‐TGF*β*1 (Abcam, ab99562), Akt (pan) (Cell Signaling Technology, #4691), phospho‐Akt (Cell Signaling Technology, #4060, RRID: AB_2315049), and *β*‐actin (Cell Signaling Technology, #3700).

After electrophoresis, the gel was submerged into a Coomassie brilliant blue dye solution, and the dye solution could completely cover the gel. The gel was shaken slowly on a horizontal or side shaker and dyed at room temperature for 1 h. Then, the gel was added with a proper amount of deionized water, washed three times for 5 min each time, subsequently placed in a white background, and photographed. Each experiment was replicated three times.

### Plate Colony Formation Assay

MKN45 cells were placed in a 6‐well plate at 200 cells per well and cultured for about 12 days, and the chamber (Millipore, MCHT06H48) was placed in the second, third, and fourth wells. CAFs (1 × 106) were planted on the chamber, and then IgG (Sigma Aldrich, Cat# I5381, RRID: AB_1163670) and FBN1 K672 antibodies were added into the third and fourth wells, respectively. The cells were fixed with 70% methanol and stained with Giemsa solution. The cells were considered alive when the colony exceeds 50 cells. The experiments were replicated three times.

### Binding Kinetic Analysis

Binding kinetics was performed by BLI using an Octet K2 instrument (Pall Life Sciences, Port Washington, NY). Recombinant MMP2 (Sino Biological Inc., China) was immobilized onto the amine‐reactive second‐generation biosensor with an amine coupling kit. Different concentrations of K672 polypeptide or succinylated K672 polypeptide (R&D system) were added to the mobile phase to detect the correlation between immobilized and flowing proteins. The detection condition was 20 × 10^−3^ m HEPES (pH 7.5, 150 × 10^−3^ m NaCl, 0.05% [v/v] Tween‐20, and 1 × 10^−3^ m MgCl2 [or 5 × 10^−3^ m EDTA]). Then, the binding kinetics was analyzed using the ForteBio Data Analysis 9.0 software.

### Spheroid Cell Invasion Assay

A 96‐well 3D spherical cell invasion kit (Trevigen, MD, USA) was used to perform the spherical cell invasion experiment. Cell culture was carried out following the recommendations of the manufacturer. The collected cells were resuspended in spheroid formation ECM, centrifuged at 200 × g for 3 min at room temperature, and cultured at 37 °C for 72 h. Fifty microliters of invasive matrix was added to each well on ice and centrifuged at 300 × *g* for 5 min at 4 °C. The petri dish was incubated at 37 °C for 1 h to stimulate gel formation. Then, cell culture medium (100 µL per well) containing chemical attractants and invasion‐modulating compounds was added and incubated at 37 °C for 3–6 days. A 4× objective was used to photograph the spheres in each hole every 24 h. ImageJ software (http://rsb.info.nih.gov/ij/, ImageJ, RRID: SCR_003070) was used to analyze the image and assess the invasiveness of 3D cultured cells. Each experiment was replicated three times.

### Zebrafish Cell Line‐Derived Xenograft (zCDX) Model

MKN45 cells were labeled with red fluorescence, while CAFs were not labeled. Before injection, the two kinds of cells were mixed according to 1:1, and the two kinds of cell mixture were respectively administered in vitro (IgG and K672suc antibody), and then co‐injected into zebrafish embryos.^[^ ^45^ ^]^ The assay was divided into four groups: MKN45 cells, MKN45 and CAFs, IgG‐treated MKN45 and CAFs, and K672suc antibody‐treated MKN45 and CAFs. The cells were injected into the yolk sac of the embryo 2 days postfertilization. Three days after injection, the proliferation of MKN45 cells in each group was quantified. ImageJ was used to analyze the area of metastatic tumor cells.

### Bioinformatics Analysis

Publicly available transcriptomic cohorts for various kinds of cancer and normal tissues were searched from TCGA and Genotype‐Tissue Expression. RNA gene expression was expressed as transcripts per million reads and not exons per thousand base fragments per million reads. Clinical data for stomach adenocarcinoma were downloaded from LinkedOmics.^[^ ^46^ ^]^ The data above were analyzed with R (version 3.5.2). Correlation analyses were conducted using Pearson correlation. RNA‐seq data (HTSeq‐FPKM data) were obtained from the TCGA database (https://portal.gdc.cancer.gov/) STAD projects. The data was calculated using FPKM values log2‐transformed. Visualization was carried out using the R package ggplot2.

### Immunofluorescence Staining

The CAFs were evenly spread on 6‐well plates, treated with or without MKN45 cells for 48 h, placed in 4% paraformaldehyde for 15 min, washed thrice for 5 min each with PBS, and permeated with 0.1% Triton X‐100 for 10 min. Then, the cells were blocked with 3% BSA at 37 °C for 1 h, incubated with primary anti‐MMP‐2 antibody (Cell Signaling Technology, Cat. # 40994, RRID: AB_2799191) overnight at 4 °C, and rinsed thrice with PBS for 5 min each. The cells were incubated with the corresponding secondary antibody for 1 h at 37 °C, washed with 0.05% Tween, and stained with DAPI (Sigma, F6057). Finally, the cells were observed and photographed under a microscope (Olympus URFL‐T, IX51, Japan). The experiments were replicated three times.

### Enzyme‐Linked Immunosorbent Assay (ELISA)

The four polypeptides were applied to a 96‐well enzyme plate overnight at 4 °C, then washed with PBS five times for 3 min each, blocked for 2 h with 5% BSA, incubated for 2 h with MMP2 protein, and shaken several times. Afterward, the polypeptides were washed again with PBS five times for 3 min each. MMP2 monoclonal antibody (Cell Signaling Technology, Cat# 40994, RRID: AB_2799191) was incubated for 1 h with shaking. The plate was washed with PBS five times for 3 min each, incubated with the secondary antibody (anti‐rabbit IgG, HRP‐linked antibody; Cell Signaling Technology, Cat# 7074, RRID: AB_2099233) for 30 min, and shaken several times. Afterward, the plate was washed with PBS five times for 3 min each and added with 3,3′ 5,5′‐tetramethylbenzidine chromogenic solution (Sigma‐Aldrich, T0440) for 5 min. The reaction was stopped with 2 m HCl, and the absorbance was read at 405 nm. All the manipulations were carried out at room temperature.

ELISA kit (Thermo Fisher Scientific, Invitrogen, MA, USA) was used to detect MMP2 and FBN1 in the cell culture supernatant. Each experiment was repeated at least three times. Each experiment was replicated three times.

### Chromatin Immunoprecipitation (ChIP)

The SimpleChIP Enzymatic ChIP Kit (Cell Signaling Technology, MA, USA) was used in ChIP detection. First, the cell lysate was prepared. Then, micrococcal nuclease was used to cleave the chromatin into a size of 150–900 bp and enriched chromatin with the magnetic beads of HIF‐1*α* or isotype IgG. The cross‐links for the enriched and input DNA were enriched and washed with NaCl and proteinase K before purification. Lastly, polymerase chain reaction (PCR) was performed to analyze the specific sequences of the immunoprecipitated and input DNAs at the promoter region, upstream of MMP2 (Forward primer: 5′CTGACCCCCAGTCCTATCTGCC3′, reverse primer: 5′TGTTGGGAACGCCTGACTTCAG3′).

### Statistical Analysis

GraphPad Prism 6.0 software (RRID: SCR_002798) was used for statistical analysis. Student's *t* test and Chi‐square test were performed for continuous variables and categorical variables, respectively. Kaplan–Meier and Gehan–Breslow–Wilcoxon tests were used to calculate OS. All the values in the text and graphs are based on mean ± standard deviation (SD). *p* < 0.05 indicated a significant difference. The sample size (*n*) values are provided in the relevant figures. The experiments were repeated at least three times.

### RNA Interference Analysis

FBN1 shRNA, MMP2 shRNA, and corresponding control shRNA plasmids were purchased from Shanghai Genechem Co., Ltd. (Shanghai, China). The shRNA sequence for FBN1 is CCTGCATTGATAACAATGAAT (sigma, USA). The shRNA sequences for MMP2 are as follows: (#1) GCTGAAAGATACCCTCAAGAA, (#2) CCGGGATAAGAAATATGGATT, (#3) GCTGTGTTCTTCGCAGGGAAT. The transfections were performed with Lipofectamine 3000. The protein samples were collected for Western blot detection after transfection for 48 h.

### Xenograft Model

Male nude mice (4‐6 weeks old) were purchased from Model Animal Research Center of Nanjing University (Nanjing, Jiangsu, China). All animal studies were approved by the Nanjing Medical University Ethics Review Board. Approximately 5 × 10^6^ MKN45 cells with various treatment were subcutaneously injected into the nude mice. Groups are MKN45, MKN45 with CAFs (1 ×10^6^), MKN45 with CAFs, and IgG or K672suc antibody(1 µg µL^−1^, 5 µL). Rescue experiments are grouped as MKN45^shRNA control^, MKN45^shRNA MMP2^ with PI3K inhibitor (PI3K/AKT‐IN‐1, Cat# HY‐144806, MedChemExpress, Shanghai, China), MKN45^shRNA control^ with CAFs, MKN45^shRNA MMP2^ with CAFs and PI3K inhibitor (*n* = 3). The tumor tissues were removed after 2 weeks, and the mice were euthanized. Tumor volume was calculated as width × length × (width + length)/2.

### Cell Invasion Assay

The cell invasion assay was performed in a 24‐well Transwell Chamber (Costar, Corning, NY, USA) coated with Matrigel (BD Pharmingen, San Jose, CA, USA). MKN45 cells (2 × 10^5^ /200 µL) with different treatment were cultured in the upper compartment in serum‐free medium. Groups are MKN45, MKN45 with CAFs (4 ×10^4^), MKN45 with CAFs and K672suc antibody(1 µg µL^−1^,5 µL). In the lower compartment, 10% complete medium was added. After incubation at 37 °C for 24 h, the cells were fixed with 4% paraformaldehyde, stained by crystal violet, and then photographed under a microscope.

### Luciferase Reporter Assay

The wild‐type or mutated sequences within the predicted binding sites of HIF‐1*α* were synthesized, inserted into a luciferase reporter plasmid (Genechem, Shanghai, China), and transfected into 293T cells. After 24 h, the cells were treated with DHT at 100 × 10^−9^ m for 24 h. The luciferase activity was normalized to Renilla luciferase activity. Hif‐1*α* promoter sequence was extracted from https://www.ncbi.nlm.nih.gov/nuccore/NM_001530.4/ (NM_001530.4‐promoter). MMP2 transcript was extracted from https://www.ncbi.nlm.nih.gov/nuccore/NM_004530.6 (NM_004530).

### Co‐Immunoprecipitation

The co‐immunoprecipitation (co‐IP) assay was performed as described before.^[^ ^47^ ^]^ In brief, cells were lysed in IP buffer (20 × 10^−3^ m Tris, pH 7.5, 150 × 10^−3^ m NaCl, 1% Triton X‐100, and 1 × 10^−3^ m EDTA) containing protease inhibitors (Roche Applied Science, Mannheim, Germany) on ice for 30 min. Subsequently, the cells were centrifuged, and the supernatant was collected, followed by incubation with primary antibodies and GammaBind Plus Sepharose (#17088601; GE Healthcare, Logan, UT, USA) with gentle rocking overnight at 4 °C. The next day, the mixture was pelleted, washed six times with cold 1× IP buffer, and then analyzed by Western blot.

## Conflict of Interest

The authors declare no conflict of interest.

## Authors Contribution

X.W., X.S., and H.L. contributed equally to this work. The experiments were designed by J.W., X.W., and C.Z. The research was performed by X.W, X.S., H.L., C.Z., and X.L. The data were analyzed by J.S. and T.Z. The manuscript was written by J.W., X.W., X.S., and C.Z.

## Data Availability

The data that support the findings of this study are available from the corresponding author upon reasonable request.
